# Bone quality in total shoulder arthroplasty: a prospective study correlating computed tomography Hounsfield units with thumb test and fracture risk assessment tool score

**DOI:** 10.1016/j.jseint.2023.03.012

**Published:** 2023-04-12

**Authors:** Alexander Hayden, Eric J. Cotter, Terah Hennick, Scott Hetzel, John Wollaeger, Scott Anderson, Brian F. Grogan

**Affiliations:** a Department of Orthopedics and Rehabilitation, University of Wisconsin School of Medicine and Public Health, Madison, WI, USA; b Department of Biostatistics and Medical Informatics, University of Wisconsin School of Medicine and Public Health, Madison, WI, USA

**Keywords:** Bone quality, Humerus, Shoulder arthroplasty, Computed tomography, Hounsfield units, Fracture risk assessment tool

## Abstract

**Background:**

To evaluate if Hounsfield units (HU) measured on preoperative computed tomography (CT) scans at the anatomic neck of the proximal humerus correlates with intraoperative findings of the “thumb test” in assessment of bone quality in shoulder arthroplasty patients.

**Methods:**

Primary anatomic total shoulder and reverse total shoulder arthroplasty patients from 2019-2022 with an available preoperative CT scan of the operative shoulder were prospectively enrolled at a single center with 3 surgeons who perform shoulder arthroplasty. The “thumb test” was performed intraoperatively; a positive test signified “good bone.” Demographic information, including prior dual x-ray absorptiometry scans, was extracted from the medical record. HU at the cut surface of the proximal humerus were calculated, as was cortical bone thickness on preoperative CT. Fracture risk assessment tool (FRAX) scores were calculated for 10-year risk of osteoporotic fracture.

**Results:**

A total of 149 patients were enrolled. Mean age was 67.6 ± 8.5 years with 69 (46.3%) being males. Patients with a negative thumb test were significantly older (72.3 ± 6.6 vs. 66.5 ± 8.6 years; *P* < .001) than those with a positive thumb test. Males were more likely to have a positive thumb test than females (*P* = .014). Patients with a negative thumb test had significantly lower HUs on preoperative CT (16.3 ± 29.7 vs. 51.9 ± 35.2; *P* < .001). Patients with a negative thumb test had a higher mean FRAX score (14.1 ± 7.9 vs. 8.0 ± 4.8; *P* < .001). Receiver operator curve analysis was performed to identify a cut-off value for CT HU of 36.67, above which the thumb test is likely to be positive. Furthermore, receiver operator curve analysis also identified optimal cut-off values for 10-year risk of fracture by FRAX score of 7.75 HU, below which the thumb test is likely to be positive. Fifty patients were at high risk based on FRAX and HU; surgeons classified 21 (42%) as having “poor bone” quality through a negative thumb test. High-risk patients had a negative thumb test 33.8% (23/68) and 37.1% (26/71) of the time for HU and FRAX, respectively.

**Conclusions:**

Surgeons are poor at identifying suboptimal bone quality at the anatomic neck of the proximal humerus based on intraoperative thumb test when referencing against CT HU and FRAX scores. The objective measures of CT HU and FRAX scoring may be useful metrics to incorporate into surgeons’ preoperative plans for humeral stem fixation using readily available imaging and demographic data.

There have been significant advances to shoulder arthroplasty component design over the past 20 years, including the introduction of “stemless” or canal sparing implants in 2004.^4^^,^^12^ The goal of total shoulder arthroplasty (TSA) is to restore, as closely as feasible, native joint mechanics and soft-tissue tension while minimizing adverse effects on the proximal humerus. Early generation implants were long-stemmed components that had several disadvantages, including stress shielding of proximal humeral bone, difficulty restoring native joint mechanics in cases with existing proximal humeral deformities, periprosthetic humerus fractures including the greater tuberosity, and challenging revision arthroplasty procedures that may require osteotomies for stem extraction.^4^^,^^19^ As a result, short-stemmed and “stemless” implants have been designed to create a functional, stable joint while minimizing adverse effects of traditional long-stemmed implants. Furthermore, these implants are less affected by variable proximal humeral anatomy that can alter joint mechanics based on stem placement.^4^^,^^15^ Short-term clinical results have been favorable, with several authors noting no difference in patient-reported outcome measures, complications, and failure rates between stemless and stemmed components while also reporting shorter operative time and decreased blood loss in stemless cases.^14^^,^^15^^,^^18^ Many stemless components rely on press-fit into the metaphysis for fixation following an osteotomy at the level of the anatomic neck. Adequate metaphyseal bone quality is necessary to achieve strong fixation and avoid acute or long-term secondary loosening.^18^^,^^20^ A finite element analysis investigation reported micromotion in 99% of implanted stemless implants when loaded in abduction, which emphasizes the importance of careful patient selection for use of these implants.^10^

Historically, proximal humeral metaphyseal bone quality has been assessed using preoperative radiographs and intraoperative visualization evaluating for cysts as well as the use of the “thumb test” as described by Churchill et al.^5^ The thumb test is performed intraoperatively when the surgeon presses his or her thumb into the proximal metaphyseal bone following the anatomic neck cut. If minimal force can impact the bone then it is not deemed acceptable for stemless components.^5^ The thumb test is subjective and variability may exist depending on the individual surgeon’s opinion of what constitutes “minimal force” and where exactly a surgeon presses (calcar peripheral bone or central over the canal).

Bone density can also be evaluated using dual x-ray absorptiometry (DXA) scans, which provide surgeons objective preoperative data regarding expected intraoperative bone quality. However, DXA scans are not always available in shoulder arthroplasty patients. A patient’s fracture risk assessment tool (FRAX) score, intended for postmenopausal women and men aged 50 years and more to estimate percent risk of sustaining a fracture over a 10-year time frame,^6^^,^^11^ is a measure that augments the DXA data and may enhance the surgeon’s assessment of bone quality. In recent years, opportunistic measurement of Hounsfield units (HU) on computed tomography (CT) scans has also been shown to be a reliable and accurate way of assessing bone quality, and it can be used as a screening tool to identify those with low bone mineral density.^2^^,^^9^ HU are a relative quantitative measurement of radiodensity of tissues on CT scan. Air is defined as −1000 HU, distilled water at standard temperature and pressure is 0, cortical bone is roughly 1000, and metals can be 3000s or more HU.^8^ CT scans are routinely obtained at many centers prior to TSA for preoperative planning purposes. These CT scans have the potential to provide surgeons with an assessment of bone quality preoperatively by measurement of HU and allow for planning of a stemless or stemmed component.^13^

The primary purpose of the study was to evaluate if HU measured on preoperative CT scans at the anatomic neck of the proximal humerus correlate with intraoperative “thumb test” results in assessment of bone quality in shoulder arthroplasty patients. A secondary aim was to determine if proximal humerus cortical thickness correlates with proximal humerus HU and thumb test results. The hypothesis of this study was that the ability of surgeons to subjectively determine proximal humeral bone quality would correlate strongly with assessment of bone quality as measured in HU at the anatomic neck on preoperative CT scans.

## Methods

Following institutional review board approval (#2019-1217), consecutive patients scheduled to undergo primary anatomic TSA or primary reverse total shoulder arthroplasty (rTSA) at a single academic institution from December 26, 2019 through April 1, 2022 were prospectively enrolled. Patients routinely underwent a noncontrast CT scan of the operative shoulder preoperatively for templating purposes. Patients were included if they underwent a primary anatomic or rTSA for any diagnosis not related to fracture or avascular necrosis, as these diagnoses may affect proximal humeral bone quality. Patients were excluded if they did not have a preoperative CT scan obtained within 12 months of surgery that was available for review in our institution’s radiology picture archiving and communication system, had a benign or malignant tumor of the proximal humerus, were actively being treated for cancer, were immunosuppressed, had an active infection, or had a diagnosis of inflammatory arthropathy. Revision cases were also excluded.

### Patient demographics and surgical information

Patient demographic information was extracted from the electronic medical record, including age at time of surgery, diagnosis, sex, laterality, body mass index, history of previous ipsilateral shoulder surgery, medical comorbidities, smoking status, alcohol use, history of proximal humerus fractures, and number of prior fractures of any kind. Furthermore, information regarding prior DXA scans, FRAX score, diagnosis of osteopenia or osteoporosis, active and prior pharmacologic treatment for low bone density, and surgical procedure and implants used was recorded.

### Computed tomography bone density assessment

**Figure 1:**
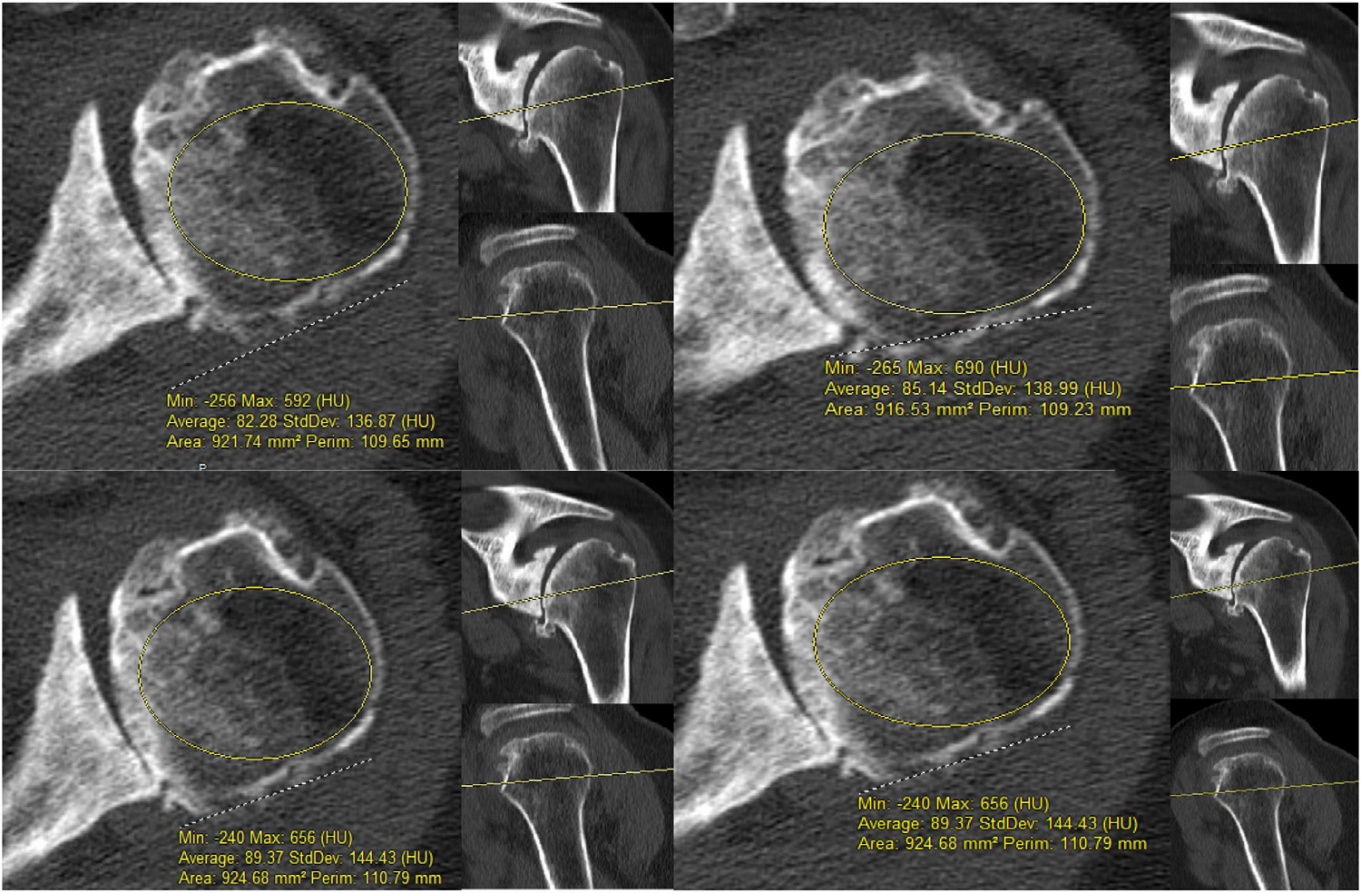
Computed tomography (CT) Hounsfield unit (HU) measurement. Four consecutive axial slices of the symptomatic shoulder CT scan were measured. A region of interest was drawn within the humeral head as outlined in this example. Average HU values were recorded. Two independent authors performed all measurements.

The standard protocol for preoperative shoulder CT scans at this institution includes 1.25 mm slice thickness, 140 kV, 100 mAs, pitch 0.52, image resolution of 512 × 512 pixels, and rotation time of 0.70 seconds. All included patients had their preoperative CT scans reviewed in our picture archiving and communication system (Change Healthcare Enterprise Viewer; Change Healthcare, Nashville, TN, USA) by 2 independent, blinded reviewers (A.H. and T.H.). Neither reviewer had knowledge of the results of the thumb test for any patient. HU measurements were performed using the CT scans as previously reported.^9^^,^^13^ Specifically, the axial cut bisecting the center of the humeral head including part of the anatomic neck was located by using the reformatted coronal and sagittal CT images to coordinate. A best-fit circle over subchondral bone and excluding cortical bone was then calculated on 4 consecutive slices (2 cranial and 2 caudal) from the aforementioned axial cut through the anatomic neck. HU were recorded and then averaged (Fig. 1). This methodology allows for a summary of 5 mm of consecutive axial CT cuts traversing through the metaphyseal bone at the anatomic neck for a comprehensive bone health assessment and has been previously published.^9^

**Figure 2:**
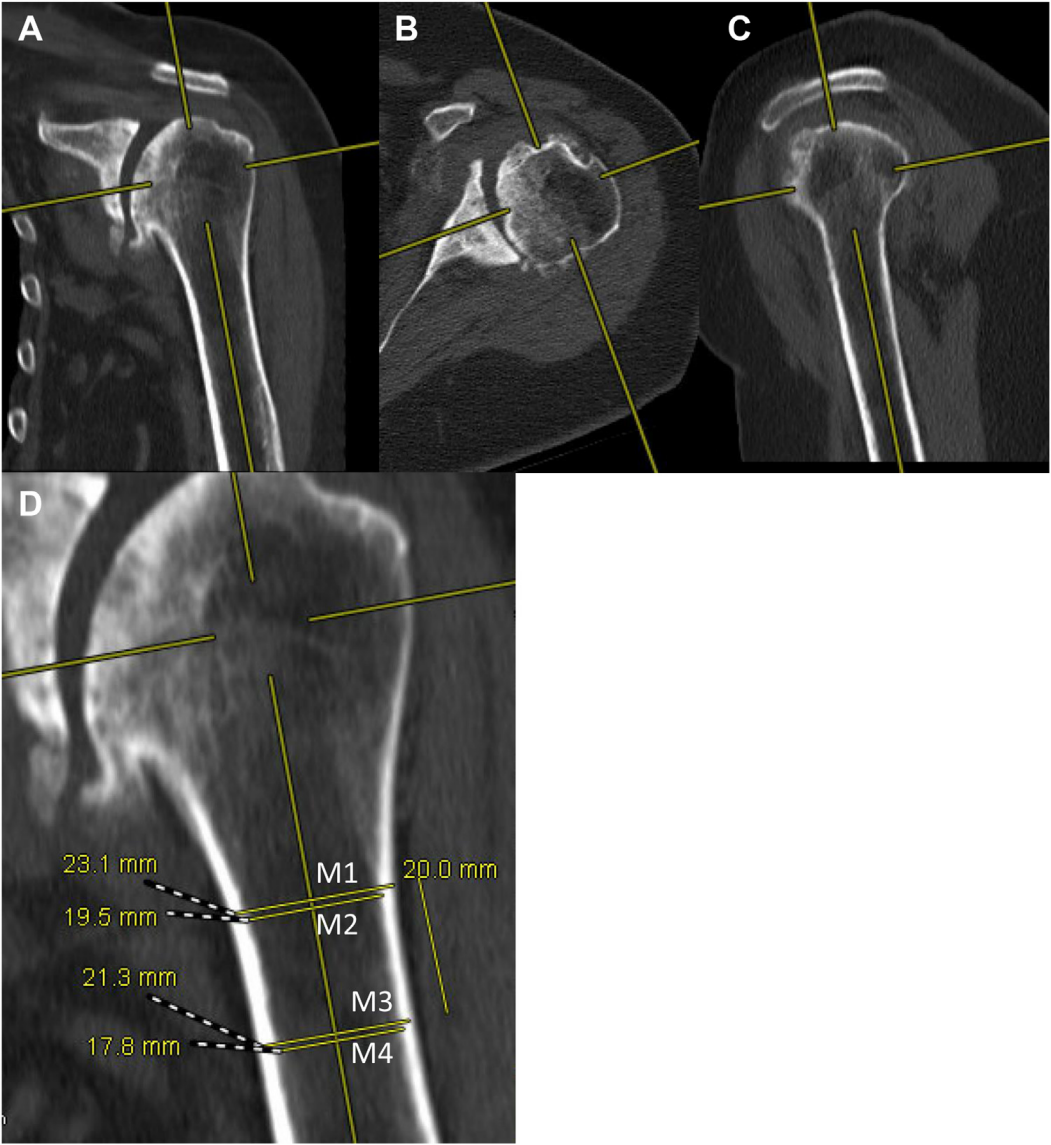
Cortical bone thickness measurement. The 3D multiplane reconstruction function was used to orient the humerus. (**A**) Demonstrates the coronal orientation, in which the anatomic axis of the humerus was identified. (**B**) Demonstrates axial orientation, centered in the humeral head and bisecting the glenoid. (**C**) Demonstrates the sagittal plane, which also defines the anatomic axis of the humerus. (**D**) Demonstrates the CBT measurement: M1/M2 was measured at the first level where medial and lateral cortices were parallel to each other. M3/M4 was measured 20 mm distal to that. M1-M4 are perpendiculars to the medial and lateral cortices. *CBT*, cortical bone thickness.

Humeral cortical thickness was measured on CT using the methodology of Schmidutz et al adapted from prior authors who first published this technique using radiographs.^16^^,^^17^^,^^24^ In brief, the first level of measurement was taken at the point where the outer medial and lateral cortical borders become parallel. A perpendicular line was then drawn from the medial outer cortex of the humerus to the lateral outer cortex of the humerus and measured with a digital caliper (M1). Next, the inner cortical diameters were measured with the digital caliper at the same level (M2). This distance of M2 was subtracted from M1 to obtain the cortical thickness proximally, which was termed C1. The second level was measured 20 mm distal to the first point using the same methodology and termed C2. The C1 and C2 values were then averaged to determine cortical bone thickness (CBT) average (Fig. 2).^16^^,^^21^^,^^24^ The CBT gauge measurement was determined by dividing the CBT average by the total bone diameter (width at M1) at the most proximal measurement level.^16^ These measurements were taken by the same 2 independent reviewers, who again were blinded to the results of the thumb test.

### Intraoperative bone quality assessment

Following the “head” cut of the proximal humerus, patients had their proximal humeral bone quality at the anatomic neck assessed using the “thumb test” by the attending surgeon. This was performed as described by Churchill et al.^5^ Bone that compressed with minimal force had a negative thumb test or “poor bone” quality. Conversely, bone that could not be compressed with minimal force had a positive thumb test or “good bone”. Three surgeons perform shoulder arthroplasty at the study center and participated in the study. Stemless implants are not routinely used by all surgeons at our center, and so the results of the thumb test may or may not have affected implant selection.

### Statistical analysis

An *a priori* power analysis was performed, and based on previous data, it was assumed that the study population would have a 20% rate of positive thumb test.^3^^,^^5^^,^^7^ It was also assumed that those with a positive thumb test would have HU that were on average 20 units more than those with a negative thumb test were. Finally, it was assumed that there would be a rather large 28 SD for HU measures.^9^ Based on these assumptions, 100 total subjects were needed to achieve 80% power in a two-sided *t*-test with alpha of 0.05. To account for possible error in the power analysis, the sample size was inflated to include 150 total subjects.

Collected data were summarized via mean (standard deviation), median (interquartile range), or N (%) for the full cohort and between those with positive and negative thumb test results. Demographic variables were compared between positive and negative thumb test groups by simple *t*-test, Wilcoxon rank-sum test, or Chi-square tests when appropriate. The triplicate measurements of HU and CBT were examined for intrarater and inter-rater agreement using intra-rater correlation coefficent (ICC [3,1]) and inter-rater correlation coefficient (ICC [2,1]) as defined by Shrout and Fleiss for the 2 raters that measured HU and CBT from the CT scans.^22^ Further analyses of HU were based on the average of the 2 raters’ measures. Poor inter-rater reliability was seen for CBT and further analyses were done separately for each rater using the average of the 3 scores for each rater. Comparison of HU, CBT, FRAX score, and presence of osteopenia and osteoporosis between thumb test result groups were examined first by *t*-test or Chi-square test and then in a logistic regression model that controlled for age, sex, surgeon, and TSA type as covariates. Finally, receiver operator curves (ROCs) were constructed for each continuous outcome and using Youden’s index an optimal cut-value for each outcome was calculated.^25^ Statistical analysis was performed with R for statistical computing version 4.0 (R Foundation for Statistical Computing, Vienna, Austria) with alpha set at 0.05.

## Results

One hundred and forty nine patients were enrolled after 1 patient was excluded for a CT scan more than 12 months prior to surgery. The overall mean age was 67.6 ± 8.5 years and there were 69 (46.3%) males in the cohort. Patients who had a negative thumb test intraoperatively were significantly older (72.3 ± 6.6 vs. 66.5 ± 8.6 years; *P* < .001) than those with a positive thumb test or “good bone.” Furthermore, males were more likely to have a positive thumb test than females (*P* = .014). Patients with “poor bone” based on thumb test were more likely to undergo an rTSA (*P* = .021). A complete description of demographic, comorbidity, and arthroplasty type can be found in Table I.

**Table I:** Demographics, comorbidities, and arthroplasty type for the entire cohort and subgroup by thumb test result.

Variable	Thumb test result			*P* value
	Total N = 149	Positive (n = 120)	Negative (n = 29)	
Surgeon				.124
Surgeon 1	35 (23.5%)	24 (20.0%)	11 (37.9%)	
Surgeon 2	88 (59.1%)	74 (61.7%)	14 (48.3%)	
Surgeon 3	26 (17.4%)	22 (18.3%)	4 (13.8%)	
Age (y)∗	67.6 (8.5)	66.5 (8.6)	72.3 (6.6)	<.001
Anatomic TSA	56 (37.6%)	51 (42.5%)	5 (17.2%)	.021
Diagnosis				.614
GHJ arthritis	95 (63.8%)	78 (65.0%)	17 (58.6%)	
Rotator Cuff Arthropathy	53 (35.6%)	41 (34.2%)	12 (41.4%)	
Instability	1 (0.7%)	1 (0.8%)	0 (0.0%)	
Sex, Male	69 (46.3%)	62 (51.7%)	7 (24.1%)	.014
Laterality, Left	63 (42.3%)	52 (43.3%)	11 (37.9%)	.75
BMI (kg/m^2^)	31.7 (6.0)	31.8 (5.9)	31.2 (6.7)	.614
Prior Shoulder Surgery	51 (34.2%)	40 (33.3%)	11 (37.9%)	.802
Prior Contralateral TSA	26 (17.4%)	20 (16.7%)	6 (20.7%)	.811
Prior RCR	11 (7.4%)	9 (7.5%)	2 (6.9%)	1
Hypertension	89 (59.7%)	72 (60.0%)	17 (58.6%)	1
Smoking Status				.887
Never	77 (51.7%)	62 (51.7%)	15 (51.7%)	
Former	64 (43.0%)	52 (43.3%)	12 (41.4%)	
Current	8 (5.4%)	6 (5.0%)	2 (6.9%)	
Diabetes	39 (26.2%)	35 (29.2%)	4 (13.8%)	.146
CAD	19 (12.8%)	16 (13.3%)	3 (10.3%)	1
Current or Prior Cancer Diagnosis	24 (16.1%)	20 (16.7%)	4 (13.8%)	1
Prior Fracture >50 y old	8 (5.4%)	5 (4.2%)	3 (10.3%)	.187
Prior DXA	40 (26.8%)	30 (25.0%)	10 (34.5%)	.423
Current Osteoporosis Medications				.002
None	90 (61.2%)	80 (67.8%)	10 (34.5%)	
OTC	56 (38.1%)	37 (31.4%)	19 (65.5%)	
Rx	1 (0.7%)	1 (0.8%)	0 (0.0%)	
Both	0 (0.0%)	0 (0.0%)	0 (0.0%)	
Previous Osteoporosis Medications				.021
None	115 (78.2%)	97 (82.2%)	18 (62.1%)	
OTC	31 (21.1%)	21 (17.8%)	10 (34.5%)	
Rx	0 (0.0%)	0 (0.0%)	0 (0.0%)	
Both	1 (0.7%)	0 (0.0%)	1 (3.4%)	
Rotator Cuff Intact	103 (69.1%)	87 (72.5%)	16 (55.2%)	.112
CT to Surgery (weeks)∗	7.3 (2.6-12.7)	7.8 (2.7-13.2)	3.9 (2.1-12.0)	.222

*TSA*, total shoulder arthroplasty; *GHJ*, glenohumeral joint; *BMI*, body mass index; *RCR*, rotator cuff repair; *CAD*, coronary artery disease; *OTC*, over the counter; *Rx*, prescription; *CT*, computed tomography.

∗ Reported as mean (standard deviation), N (%) or median (interquartile range).

**Figure 3:**
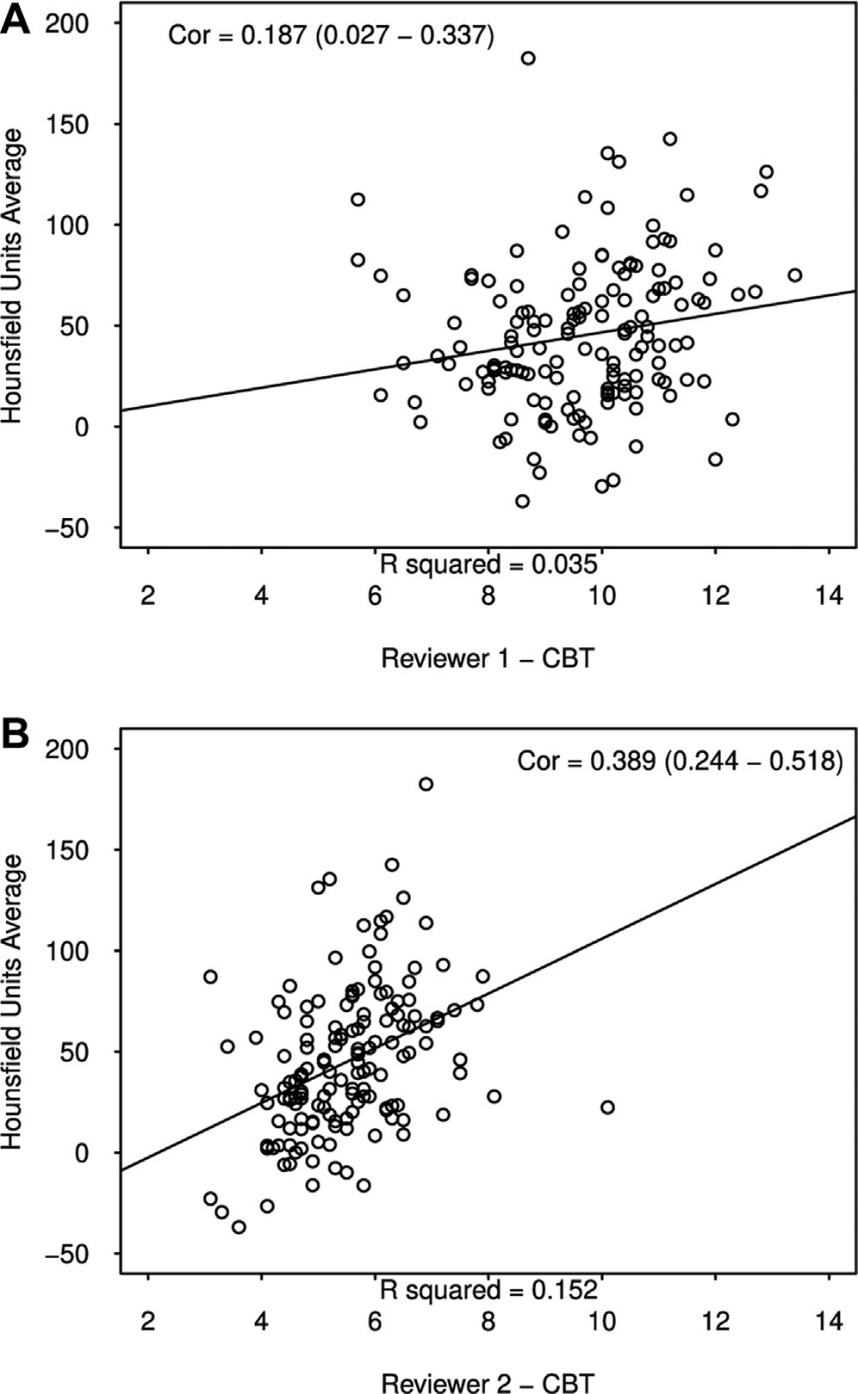
Scatterplots demonstrating the relationship between computed tomography (CT) Hounsfield unit (HU) and cortical bone thickness (CBT) for reviewers #1 and #2, respectively. (**A**) demonstrates a weak positive linear correlation because Hounsfield units on computed tomography scan with cortical bone thickness was based on reviewer #1’s measurements. (**B**) demonstrates the same relationships for reviewer #2.

Both independent reviewers demonstrated excellent reliability when making CT HU and CBT measurements with intrarater correlation coefficients >0.9 for all. The inter-rater reliability for CT HU was good with an ICC for 0.812 (0.695-0.876). The inter-rater reliability for CBT gauge was poor as demonstrated by an ICC of 0.082 (−0.038 to 0.241). Weak positive linear relationships were identified between CBT and CT HU for reviewer #1 and #2, respectively. Figure 3 contains 2 scatterplots demonstrating these relationships. Table II details the intrarater and inter-rater reliability outcomes of image analysis.

**Table II:** Inter-rater reliability of Hounsfield units and cortical bone thickness.

Variable	Intrarater∗		Inter-rater†
	Reviewer 1	Reviewer 2	
CT HU	0.978 (0.973-0.983)	0.930 (0.913-0.944)	0.812 (0.695-0.876)
CBT			0.064 (−0.034 to 0.196)
M1 and M3	0.927 (0.905-0.943)	0.957 (0.943-0.967)	
M2 and M4	0.915 (0.890-0.934)	0.939 (0.921-0.953)	
CBT Gauge			0.082 (−0.038 to 0.241)

*CT*, computed tomography; *HU*, Hounsfield unit; *CBT*, cortical bone thickness.

∗ Reported as intra-rater correlation coefficient (95% CI).

† Reported as inter-rater correlation coefficient (95% CI).

Patients who had a negative thumb test as determined by the treating surgeon had, on average, significantly lower CT HU (16.3 ± 29.7 vs. 51.9 ± 35.2; *P* = .001). In addition, patients with a negative thumb test had a higher mean FRAX score (14.1 ± 7.9 vs. 8.0 ± 4.8; *P* = .049). While only 16 patients (10.9%) of the entire cohort have previously been diagnosed with osteoporosis, 6 of them had a negative thumb test. These 6 patients represented 20.7% of the negative thumb test group. The other 10 patients made up just 8.5% of the positive thumb test group. This difference was not statistically significant, with *P* = .090 without adjusting for covariates and *P* = .689 when adjusting for covariates. A complete description of the association of independent measures of proximal humerus bone quality and corresponding thumb test result can be found in Table III.

**Table III:** Association of independent measures of proximal humerus bone quality and corresponding thumb test results.

Variable	Total N = 149	Thumb test result		*P* value	*P* value∗
		Positive (n = 120)	Negative (n = 29)		
Hounsfield Unit	45.0 (37.0)	51.9 (35.2)	16.3 (29.7)	<.001	.001
Cortical Bone Thickness - Reviewer 1	9.6 (1.5)	9.7 (1.6)	9.5 (1.2)	.534	.282
Cortical Bone Thickness - Reviewer 2	5.5 (1.1)	5.6 (1.1)	5.0 (0.8)	.001	.354
FRAX Score	9.2 (6.0)	8.0 (4.8)	14.1 (7.9)	<.001	.049
Osteopenia Diagnosis	20 (13.6%)	15 (12.7%)	5 (17.2%)	.549	.751
Osteoporosis Diagnosis	16 (10.9%)	10 (8.5%)	6 (20.7%)	.09	.689

*FRAX*, Fracture Risk Assessment Tool.

The Hounsfield unit inter-rater reliability was good and therefore the measurements were averaged between the 2 independent reviewers.

Reported as mean (standard deviation) and N (%).

∗ *P* value from logistic regression model with age, sex, surgeon, and TSA, type as covariates.

**Figure 4:**
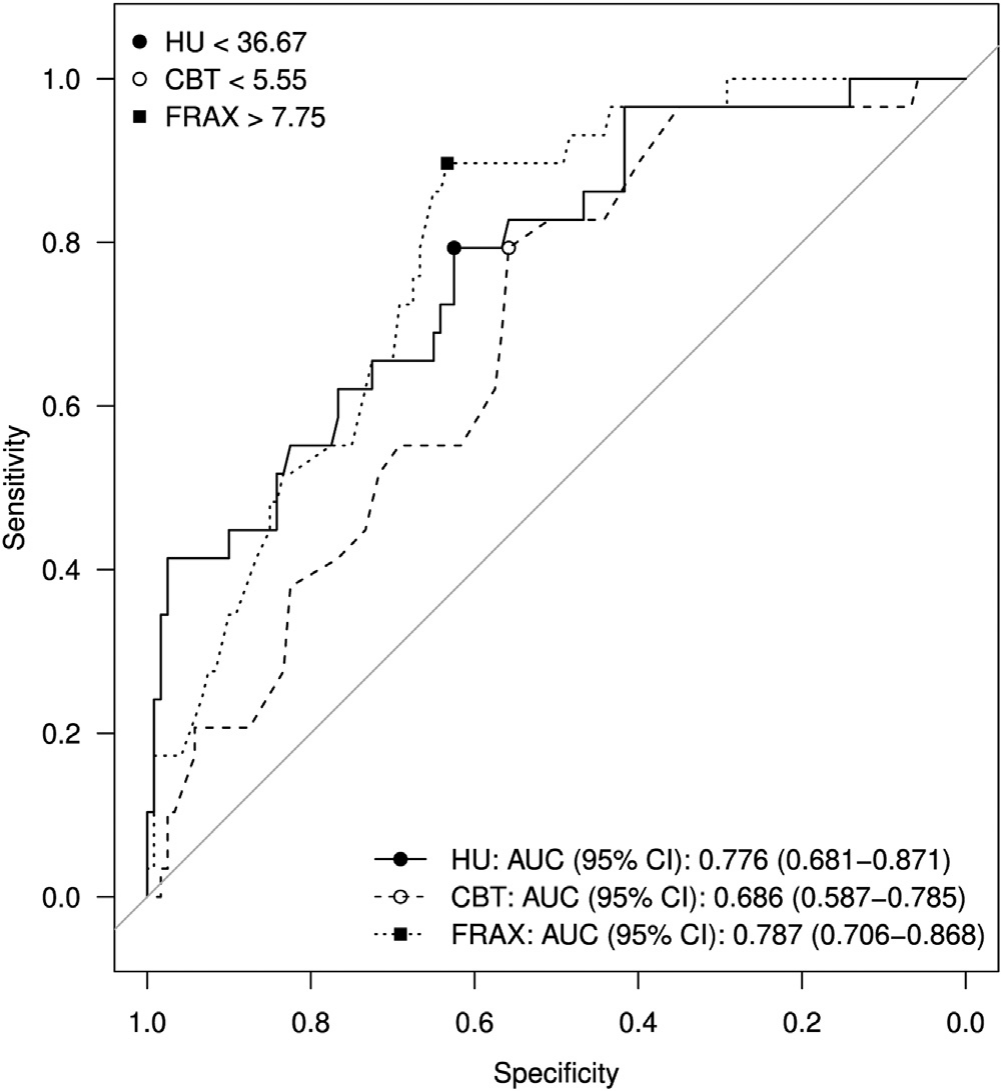
Receiver operating curve demonstrating the optimal cut off values to maximize sensitivity and specificity for computed tomography (CT) Hounsfield unit (HU), cortical bone thickness (CBT), and fracture risk assessment tool (FRAX) scores. *AUC*, area under the curve; *CI*, confidence interval.

Best-fit ROC analysis was performed to identify a cut-off value for CT HU of 36.67, above which the thumb test is likely to be positive, indicative of “good bone.” Further ROC analysis also identified optimal cut-off values for 10-year risk of fracture by FRAX score of 7.75 HU, below which the thumb test is likely to be positive. For clarity, HU > 36.67 is defined here as “Low Risk” and <36.67 as “High Risk” while FRAX score <7.75 as “Low Risk” and >7.75 as “High Risk”. Of the 50 patients who were at high risk in both variables, the surgeons classified 21 (42%) as having “poor bone” quality through a negative thumb test. Of those who were at low risk in both variables, 60 (98.3%) were classified by the treating surgeon as having “good bone” quality through a positive thumb test. Individually, those at high risk based on HU cutoff had a negative thumb test 33.8% (23/68) and those at high risk from FRAX had a negative thumb test 37.1% (26/71) of the time. Patients at low risk had a positive thumb test 92.6% (75/81) and 96.2% (76/79) of the time for HU and FRAX, respectively. These results are detailed in Table IV. These summary statistics show that the surgeons are able to correctly classify “good bone” quality based on the thumb test at a high rate but have difficulty classifying “bad bone” quality intraoperatively. Figure 4 displays a receiver operating curve with the optimal cut-off values to maximize sensitivity and specificity for CT HU, CBT, and FRAX scores.

**Table IV:** Thumb test results based on Hounsfield and FRAX cut-value risk.

	Positive (n = 120)		Negative (n = 29)	
	HU 36.67 cut		HU 36.67 cut	
FRAX Score 7.75 cut	Low Risk	High Risk	Low Risk	High Risk
Low Risk	60	16	1	2
High Risk	15	29	5	21

*HU*, Hounsfield units; *FRAX*, fracture risk assessment tool.

If either variable (higher FRAX, score or lower CT HU) is in “high risk” there is 96.6% (28/29) sensitivity for having a negative thumb test, “poor bone.”

## Discussion

We present an algorithm using preoperative shoulder CT scan and FRAX score to preoperatively predict patients with poor bone quality at the cut surface of the anatomic neck of the proximal humerus. The main findings of this study demonstrate that patients identified to have “poor bone” based on subjective thumb test intraoperatively by the treating surgeon had, on average, significantly lower CT HU at the cut surface of the anatomic neck of the humerus. Patients with a negative thumb test were also identified to have significantly higher FRAX 10-year fracture risk scores suggestive of relatively poor bone quality on a systemic level. ROC analysis identified 36.67 HU at the greatest area under the curve indicating greater likelihood of a negative thumb test in patients with lower CT HU. Importantly, this study found that surgeons were poor at identifying poor bone quality intraoperatively with use of the thumb test when referencing against a FRAX score >7.75 and CT HU < 36.67.

Churchill et al^5^ initially described the “thumb test” in 2016 in a prospective, multicenter study of 157 patients reporting on the 2-year clinical and radiographic outcomes of the Simpliciti total shoulder prosthesis in the United States. That implant is one of many “stemless” devices now in the market in the United States. An accurate, objective assessment of bone quality is important for stemless components given their reliance on nonporous humeral metaphyseal bone for stability and longevity. However, the thumb test is subject to wide variation based on the level of cut humeral surface evaluated, force used by the surgeon, and importantly, where the surgeon presses their thumb. Alidousti et al^1^ performed a spatial mapping study of the humeral head and noted that bone density increased from central to peripheral below the anatomic neck indicating the peripheral, more dense, bone is what may be of most significance when determining if a stemless device is appropriate. Many arthroplasty surgeons are now using CT scans for preoperatively templating purposes, and those same CT scans can be used to opportunistically evaluate bone quality. A preoperative understanding of bone quality is essential for implant selection, templating, and care coordination if certain implants and sizes are not routinely stocked at the care facility. The use of opportunistic CT scans in shoulder to evaluate bone quality has been described by several authors.^7^^,^^9^^,^^13^^,^^17^ Cronin et al recently noted in a prospective investigation seeking to evaluate intraoperative complications in patients undergoing TSA with low bone mineral density that surgical technique or plans were changed in 12% of cases due to bone quality. The authors also noted that surgeons were able to identify normal, healthy bone intraoperatively in 95.5% of cases but struggled at identifying poor bone (47.7% accurate). The authors did not specify how bone quality was determined intraoperatively, but CT HU were used as the gold standard comparison.^7^ The present study identified similar findings, as only 42% of patients who had both a FRAX score >7.75 and CT HU < 36.67 were reported to have a negative thumb test by the treating surgeon. Surgeons in the present study were good at identifying good bone quality base based on the 98.3% accuracy of a positive thumb in patients who had both a FRAX score <7.75 and CT HU > 36.67.

Levin et al^13^ recently published a study similar to the present one seeking to propose CT HU thresholds to allow for preoperative determination of adequate bone stock for stemless aTSA. The authors evaluated 61 patients templated to undergo a stemless aTSA based on 3-dimensional CT scan preoperatively. They evaluated the bone quality intraoperatively and found 5 patients (8.2%) with poor bone quality unfit for a stemless device at time of surgery. Those patients who received a stemmed implant were significantly older by an average of 10 years and had a lower proximal humeral HU (−1.423 +/− 17.7 vs. 78.8 +/− 52.4, *P* = .001). Based on ROC analysis in their study, a CT HU threshold of 14.4 was identified below which patients were likely to require a stemmed humeral implant. Many of these findings are consistent with those in the present study. In the present study, we found older patients were more likely to have lower CT HU and to have a negative thumb test intraoperatively. The CT HU cut-off value based on ROC analysis in our study was 36.67, which is notably higher than that reported by Levin et al.^13^ This may be due to variation in sample size as the present study has a cohort 2.5 times larger than the previous study. Levin et al used an adjunctive imaging measurement, deltoid tuberosity index (DTI), to further try to characterize proximal humeral bone quality. DTI is a metric based on preoperative radiographs where the outer cortical diameter is divided by the inner cortical diameter at the level immediately proximal to the deltoid tuberosity. DTI is similar to CBT, but without a second measurement 20 mm distal to the proximal measure. In contrast to the present study, which found poor inter-rater reliability for CBT measurements, Levin et al found DTI to be a reproducible measurement that has utility in assessing proximal humeral bone quality. Similar findings were also reported by Spross et al.^13^^,^^23^ The poor inter-rater reliability of CBT in the present study obviates its utility in determining proximal humeral bone quality preoperatively. Based on our experience, this measure should be used with caution.

Several studies have evaluated CT HU in the proximal humerus for preoperative bone assessment in surgical planning but, to our knowledge, none have incorporated the FRAX score into that assessment. The FRAX score is an easily calculated risk assessment tool using readily available demographic, DXA (if available), and social information from the medical record. Our study identified a significant association between higher FRAX scores and negative thumb tests when controlling for age, sex, surgeon, and TSA type on multivariate regression analysis. While likely an underpowered finding, there was a trend toward negative thumb test results (*P* = .09) in the 10.9% of the study cohort previously being diagnosed with osteoporosis. Including FRAX into the surgeon’s surgical implant planning may augment the CT HU data, adding another level of assurance with minimal additional preoperative effort. A more accurate understanding of bone quality preoperatively may have cost implications when considering opening additional instrument trays and maintain an appropriate inventory of implants.

### Strengths and limitations

Strengths of this study include a prospective, large sample size with *a priori* power analysis to detect a 20 HU difference between patients with a negative and positive thumb test. This study considered additional metrics including FRAX score, CBT, and current osteopenia or osteoporosis diagnosis and treatment in bone quality assessment. This study is not without limitations. We measured the HU on preoperative shoulder CT scans using 3 consecutive slices that were consistent between aTSA and rTSA patients. Clinically, the humeral neck cut may be slightly more distal in the setting of rTSA. Therefore, the area of HU measurement may be just proximal to the surface that was intraoperatively assessed. Finally, the exact location and the force used for the thumb test are undoubtedly variable.

## Conclusion

Surgeons are poor at identifying suboptimal bone quality at the anatomic neck of the proximal humerus based on intraoperative thumb test when referencing against CT HU and FRAX scores. A CT HU cut-off value of 36.67 and FRAX score cut-off of 7.75 were identified as thresholds in determining poor bone quality. CBT had poor inter-rater reliability and may not be a useful metric to determine proximal humerus bone quality. The objective measures of CT HU and FRAX scoring may be helpful metrics to incorporate into a surgeon’s preoperative plan for humeral fixation using readily available imaging and demographic data.

## Disclaimers

Funding: No funding was disclosed by the authors.

Conflicts of interest: The authors, their immediate families, and any research foundation with which they are affiliated have not received any financial payments or other benefits from any commercial entity related to the subject of this article.
